# Supplementary material for: The Level of Remnant Cholesterol and Implications for Lipid-Lowering Strategy in Hospitalized Patients with Acute Coronary Syndrome in China: Findings from the Improving Care for Cardiovascular Disease in China—Acute Coronary Syndrome Project
Source: Metabolites. 2022 Sep 24;12(10):898. doi: 10.3390/metabo12100898 (PMC9607962; doi:10.3390/metabo12100898)
Supplement: Supplementary file 1 [file metabolites-12-00898-s001.zip › metabolites-1899309-supplementary.pdf]

The level of remnant cholesterol and implications for lipid-lowering strategy in hospitalized patients with acute coronary syndrome in China: Findings from the Improving Care for Cardiovascular Disease in China-Acute Coronary Syndrome Project

Supplementary Materials

Supplementary Table S1. Quintile cut-off value and proportion of elevated remnant cholesterol levels of acute coronary syndrome patients with different LDL-C/non-HDL-C levels

	1st quintile (mmol/L)	2nd quintile (mmol/L)	Median (mmol/L)	3rd quintile (mmol/L)	4th quintile (mmol/L)	RC ≥1.0 mmol/L (%)
**LDL-C level (mmol/L)**						
LDL-C<1.4	0.3	0.5	0.6	0.7	1.2	24.4
1.4≤ LDL-C＜1.8	0.3	0.5	0.5	0.6	0.8	13.3
1.8≤ LDL-C＜2.6	0.3	0.5	0.6	0.7	0.9	16.0
LDL-C≥2.6	0.4	0.6	0.7	0.8	1.0	21.7
**non-HDL-C (mmol/L)**						
non-HDL-C <2.6	0.3	0.4	0.4	0.5	0.6	2.9
2.6≤ non-HDL-C＜3.4	0.3	0.5	0.6	0.6	0.8	9.6
3.4≤ non-HDL-C＜4.1	0.4	0.6	0.7	0.8	1.0	21.7
non-HDL-C ≥4.1	0.6	0.8	0.9	1.1	1.4	44.3

LDL-C, low-density lipoprotein cholesterol; non-HDL-C, non-high-density lipoprotein cholesterol; RC, remnant cholesterol.

Supplementary Table S2. Quintile cut-off value and proportion of elevated LDL-C levels of acute coronary syndrome patients with different non-HDL-C levels

non-HDL-C (mmol/L)	1st quintile (mmol/L)	2nd quintile (mmol/L)	Median (mmol/L)	3rd quintile (mmol/L)	4th quintile (mmol/L)	LDL-C ≥1.4 mmol/L (%)	LDL-C ≥1.8 mmol/L (%)
non-HDL-C <2.6	1.4	1.6	1.7	1.8	2.0	76.9	41.0
2.6≤ non-HDL-C＜3.4	2.1	2.3	2.4	2.5	2.7	98.5	95.0
3.4≤ non-HDL-C＜4.1	2.7	2.9	3.0	3.1	3.3	99.1	98.1
non-HDL-C≥4.1	3.3	3.6	3.8	3.9	4.4	98.8	98.0

LDL-C, low-density lipoprotein cholesterol; non-HDL-C, non-high-density lipoprotein cholesterol.

Supplementary Table S3. List of participating hospitals

	Hospitals	Territories	Provinces	City	Investigator
1	Yangzhou First People's Hospital	Eastern China	Jiangsu	Yangzhou	Aihua Li
2	Shanxi Cardiovascular Hospital	Northern China	Shanxi	Taiyuan	Bao Li
3	Nanjing Drum Tower Hospital, The Affiliated Hospital of Nanjing University Medical School	Eastern China	Jiangsu	Nanjing	Biao Xu, Guangshu Han
4	Hainan General Hospital	Southern China	Hainan	Haikou	Bin Li
5	The Second Hospital of Jilin University	Northeast China	Jilin	Changchun	Bin Liu
6	Shanghai Jingan District Shibei Hospital	Eastern China	Shanghai	Shanghai	Bin Wang
7	Guangyuan Central Hospital	Northwest China	Sichuan	Guangyuan	Bing Fu
8	The 2nd Affiliated Hosiptal of Harbin Medical University	Northeast China	Heilongjia ng	Harbin	Bo Yu
9	Hospital 463 of Chinese People's Liberation Army	Northeast China	Liaoning	Shenyang	Bosong Yang
10	The Central Hospital of Mianyang	Northwest China	Sichuan	Mianyang	Caidong Luo
11	The Ninth Hospital Affiliated to Shanghai Jiaotong University School of Medicine	Eastern China	Shanghai	Shanghai	Changqian Wang
12	Zhangzhou Municipal Hospital of Fujian Province	Eastern China	Fujian	Zhangzhou	Changyong Liu
13	Shimen People's Hospital	Central China	Hunan	Changde	Chuanliang Liang
14	Henan Provincial People's Hospital	Central China	Henan	Zhengzhou	Chuanyu Gao
15	Shanxi Provincial People's Hospital	Northern China	Shanxi	Taiyuan	Chunlin Lai
16	Xihua County People's Hospital	Central China	Henan	Zhoukou	Chuntong Wang
17	Liaocheng People's Hospital	Eastern China	Shandong	Liaocheng	Chunyan Zhang
18	Yancheng Third People's Hospital	Eastern China	Jiangsu	Yancheng	Chunyang Wu
19	Quyang Renji Hospital	Northern China	Hebei	Baoding	Congliang Zhang

20	Xinqiao Hospital, Third Military Medical University	Southwest China	Chongqing	Chongqing	Cui Bin, Lan Huang
21	The Second Xiangya Hospital of Central South University	Central China	Hunan	Changsha	Daoquan Peng
22	The Central Hospital of Panzhihua	Northwest China	Sichuan	Panzhihua	Dawen Xu
23	China Meitan General Hospital	Northern China	Beijing	Beijing	Di Wu
24	Xiantao First People's Hospital	Central China	Hubei	Xiantao	Dongmei Zhu
25	Chest Hospital of Xinjiang Uygur Autonomous Region	Northwest China	Xinjiang	Urumchi	Dongsheng Chai
26	Beian First People's Hospital	Northeast China	Heilongjiang	Heihe	Dongyan Li
27	The 309th Hospital of Chinese People's Liberation Army	Northern China	Beijing	Beijing	Fakuan Tang, Jun Xiao
28	Baiyin Cite Center Hospital	Northwest China	Gansu	Baiyin	Fang Zhao
29	Deqing People's Hospital	Eastern China	Zhejiang	Huzhou	Fangfang Huang
30	Dunhua City Hospital	Northeast China	Jilin	Yanbian	Fanju Meng
31	Suizhou Central Hospital	Central China	Hubei	Suizhou	Fengwei Li
32	Binyang People's Hospital	Southern China	Guangxi	Nanning	Fudong Gan
33	The First Hospital of Qiqihaer City	Northeast China	Heilongjiang	Qiqihaer	Gang Xu
34	The Third the People's Hospital of Bengbu	Eastern China	Anhui	Bengbu	Gengsheng Sang
35	Zhongda Hospital, Southeast University	Eastern China	Jiangsu	Nanjing	Genshan Ma
36	The First Hospital of Jiamusi	Northeast China	Heilongjiang	Jiamusi	Guixia Zhang
37	The First Affiliated Hospital of Liaoning Medical University	Northeast China	Liaoning	Jinzhou	Guizhou Tao
38	Luan County People's Hospital	Northern China	Hebei	Tangshan	Guo Li
39	Guiding People's Hospital	Southwest China	Guizhou	Qinan	Guoduo Chen
40	Haidong Ping'an District Hospital of Traditional Chinese Medicine	Northwest China	Qinghai	Haidong	Guoqin Xin

41	Xinjiang Uygur Autonomous Region People’s Hospital	Northwest China	Xinjiang	Urumchi	Guoqing Li
42	Sir Run Run Shaw Hospital, College of Medicine, Zhejiang University	Eastern China	Zhejiang	Hangzhou	Guosheng Fu
43	Zhoushan People's Hospital	Eastern China	Zhejiang	Zhoushan	Guoxiong Chen
44	Dalian Municipal Central Hospital	Northeast China	Liaoning	Dalian	Hailong Lin
45	Hebei Daming County People’s Hospital	Northern China	Hebei	Handan	Haiping Guo
46	Dongguan Changping hospital	Southern China	Guangdon g	Dongguan	Haiyun Lin
47	Renmin Hospital of Wuhan University	Central China	Hubei	Wuhan	Hong Jiang
48	Honghu People’s Hospital	Central China	Hubei	Jingzhou	Hong Liu
49	Ningxia People's Hospital	Northwest China	Ningxia	Yinchuan	Hong Luan
50	The First People's Hospital of Yunnan Province (Kunhua Hospital)	Northwest China	Yunnan	Kunming	Hong Zhang
51	The People’s Hospital Feixian	Eastern China	Shandong	Linyi	Honghua Deng
52	Beijing Friendship Hospital, Capital Medical University	Northern China	Beijing	Beijing	Hongwei Li
53	The First Affiliated Hospital of Bengbu Medical College	Eastern China	Anhui	Bengbu	Honhju Wang
54	The Central Hospital of Zhoukou	Central China	Henan	Zhoukou	Hualing Liu
55	Nanpi People’s Hospital	Northern China	Hebei	Cangzhou	Hui Dong
56	Anyang District Hospital	Central China	Henan	Anyang	Hui Liu
57	Dalian Fourth People’s Hospital	Northeast China	Liaoning	Dalian	Huifang Zhang
58	General Hospital of TISCO	Northern China	Shanxi	Taiyuan	Huifeng Wang
59	Ningbo First Hospital	Eastern China	Zhejiang	Ningbo	Huimin Chu
60	Huining People’s Hospital	Northwest China	Gansu	Baiyin	Jiabin Xi
61	Jining City Yanzhou District People’s Hospital	Eastern China	Shandong	Jining	Jian Yang

62	Dongguan People's Hospital	Southern China	Guangdong	Dongguan	Jianfeng Ye
63	Panyu Hospital of Chinese Medicine	Southern China	Guangdong	Guangzhou	Jianhao Li
64	Sichuan Provincial People’s Hospital	Northwest China	Sichuan	Chengdu	Jianhong Tao
65	Mudanjiang Cardiovascular Disease Hospital	Northeast China	Heilongjiang	Mudanjiang	Jianwen Liu
66	People’s Hospital of Wugang	Central China	Hunan	Shaoyang	JiaoMei Yang
67	Yichang Central Hospital	Central China	Hubei	Yichang	Jiawang Ding
68	Zhongda Hospital, Southeast University (Jiangbei)	Eastern China	Jiangsu	Nanjing	Jiayi Tong
69	People’s Hospital of Rongchang District	Southwest China	Chongqing	Chongqing	Jie Chen
70	Peking University First Hospital	Northern China	Beijing	Beijing	Jie Jiang
71	Ye County people’s hospital	Central China	Henan	Pingdingshan	Jie Yang
72	Qilu Hospital of Shandong University	Eastern China	Shandong	Jinan	Jifu Li
73	Affiliated Hospital of Jiangsu University	Eastern China	Jiangsu	Zhenjiang	Jinchuan Yan
74	Wuhan University of Science and Technology Hospital	Central China	Hubei	Wuhan	Jing Hu
75	Shenyang City Electricity Central Hospital	Northeast China	Liaoning	Shenyang	Jing Xu
76	Sun Yat-sen Memorial Hospital, Sun Yat-sen University	Southern China	Guangdong	Guangzhou	Jingfeng Wang
77	Yuncheng Hospital	Eastern China	Shandong	Heze	Jinglan Diao
78	Fengrun District Second People’s Hospital	Northern China	Hebei	Tangshan	Jingshan Zhao
79	The First People's Hospital of Nanning City	Southern China	Guangxi	Nanning	Jinru Wei
80	Zhangping City Hospital	Eastern China	Fujian	Longyan	Jinxing Yi
81	The First Affiliated Hospital of Fujian Medical University	Eastern China	Fujian	Fuzhou	Jinzi Su
82	Chengdu Third People’s Hospital	Northwest China	Sichuan	Chengdu	Jiong Tang

83	Guangdong General Hospital	Southern China	Guangdong	Guangzhou	Jiyan Chen
84	Heilongjiang Fujin City Central Hospital	Northeast China	Heilongjiang	Jiamusi	Jiyan Yin
85	Yantaishan hospital	Eastern China	Shandong	Yantai	Juexin Fan
86	Qingdao Municipal Hospital	Eastern China	Shandong	Qingdao	Jun Guan
87	Zhongshan Hospital Affiliated to Fudan University	Eastern China	Shanghai	Shanghai	Junbo Ge
88	Hospital of Xinjiang Production & Construction Corps	Northwest China	Xinjiang	Urumchi	Junming Liu
89	Linfen People’s Hospital	Northern China	Shanxi	Linfen	Junping Deng
90	The First People’s Hospital of Horqin District, Tongliao City	Northern China	Inner Mongolia	Tongliao	Junping Fang
91	The Military General Hospital of Beijing PLA	Northern China	Beijing	Beijing	Junxia Li
92	Longyan First Hospital	Eastern China	Fujian	Longyan	Kaihong Chen
93	Guiyang Sixth People’s Hospital	Southwest China	Guizhou	Guiyang	Kalan Luo
94	Affiliated Hospital of Guangdong Medical College	Southern China	Guangdong	Guangzhou	Keng Wu
95	Jiangxi Provincial People's Hospital	Eastern China	Jiangxi	Nanchang	Lang Ji
96	The First Affiliated Hospital of Guangxi Medical University	Southern China	Guangxi	Nanning	Lang Li
97	Tongren Hospital Affiliated to Shanghai Jiaotong University School of Medicine	Eastern China	Shanghai	Shanghai	Li Jiang
98	Huaiyang People’s Hospital	Central China	Henan	Zhoukou	Li Wei
99	Binzou City Center Hospital	Eastern China	Shandong	Binzhou	Lijun Meng
100	Anhui Provincial Hospital	Eastern China	Anhui	Hefei	Likun Ma
101	Xiangtan City Central Hospital	Central China	Hunan	Xiangtan	Lilong Tang
102	Tangshan City Fengrun District People’s Hospital	Northern China	Hebei	Tangshan	Lin Wang
103	The First Hospital of Haerbin City	Northeast China	Heilongjiang	Harbin	Lin Wei

104	The First Affiliated Hospital of Zhengzhou University	Central China	Henan	Zhengzhou	Ling Li
105	Xijing Hospital	Northwest China	Shaanxi	Xi'an	Ling Tao
106	Yiniang Hospital	Southwest China	Yunnan	Kunming	Liqiong Yang
107	The Affiliated Hospital of Guizhou Medical University	Southwest China	Guizhou	Guiyang	Lirong Wu
108	Central Hospital Affiliated to Shenyang Medical College	Northeast China	Liaoning	Shenyang	Man Zhang, Kaiming Chen
109	Hepu People’s Hospital	Southern China	Guangxi	Beihai	Meisheng Lai
110	First Affiliated Hospital of the People's Liberation Army General Hospital	Northern China	Beijing	Beijing	Miao Tian
111	Yanting People’s Hospital	Southwest China	Sichuan	Mianyang	Mingcheng Bai
112	The Second People's Hospital of Yunnan Province	Southwest China	Yunnan	Kunming	Minghua Han
113	Haikou People's Hospital	Southern China	Hainan	Haikou	Moshui Chen
114	Geological Mining Hospital of Hunan Province	Central China	Hunan	Changsha	Naiyi Liang
115	The Eight Affiliated Hospital, Sun Yat-sen University	Southern China	Guangdong	Guangzhou	Nan Jia
116	The Central Hospital of Xuzhou	Eastern China	Jiangsu	Xuzhou	Peiying Zhang
117	The Second hospital of Dalian Medical University	Northeast China	Liaoning	Dalian	Peng Qu
118	The second people’s hospital of Mengcheng	Eastern China	Anhui	Bozhou	Pengfei Zhang
119	Fuqing Cite Hospital	Eastern China	Fujian	Fuqing	Ping Chen
120	The First Affiliated Hospital of Liaoning University of Traditional Chinese Medicine	Northeast China	Liaoning	Shenyang	Ping Hou
121	Gansu Provincial Hospital	Northwest China	Gansu	Lanzhou	Ping Xie
122	Beijing Tsinghua Changgung Hospital	Northern China	Beijing	Beijing	Ping Zhang
123	The First Affiliated Hospital of Henan University of Science and Technology	Central China	Henan	Luoyang	Pingshuan Dong
124	Guizhou Provincial People's Hospital	Northwest China	Guizhou	Guiyang	Qiang Wu

125	The First Affiliated Hospital of Xiamen University	Eastern China	Fujian	Xiamen	Qiang Xie
126	Chenzhou First People's Hospital	Central China	Hunan	Chenzhou	Qiaoqing Zhong
127	Lujiang People’s Hospital	Eastern China	Anhui	Hefei	Qichun Wang
128	Yuzhou City Central Hospital	Central China	Henan	Xuchang	Qinfeng Su
129	People’s Hospital of Qinghai Province	Northwest China	Qinghai	Xining	Rong Chang
130	Quanzhou First Hospital	Eastern China	Fujian	Quanzhou	Rong Lin
131	Baotou City Center Hospital	Northern China	Inner Mongolia	Baotou	Ruiping Zhao
132	Affiliated Hospital of Ningxia Medical University	Northwest China	Ningxia	Yinchuan	Shaobin Jia
133	Beijing Anzhen Hospital, Capital Medical University	Northern China	Beijing	Beijing	Shaoping Nie
134	Wuzhou People's Hospital	Southern China	Guangxi	Wuzhou	Shaowu Ye
135	North Jiangsu People's Hospital	Eastern China	Jiangsu	Yangzhou	Shenghu He
136	People’s Hospital of Bozhou District	Southwest China	Guizhou	Zunyi	Shengyong Chen
137	Shanghai Sixth People's Hospital	Eastern China	Shanghai	Shanghai	Shixin Ma
138	The Central Hospital of Jilin	Northeast China	Jilin	Changchun	Shuangbin Li
139	The First Hospital of Handan	Northern China	Hebei	Handan	Shuanli Xin
140	The Fourth Affiliated Hospital Zhejiang University School of Medicine	Eastern China	Zhejiang	Yiwu	Shudong Xia
141	Nenjiang People’s Hospital	Northeast China	Heilongjiang	Heihe	Shuhua Zhang
142	Duzishan Petrochemical Hospital	Northwest China	Xinjiang	Karamay	Shuqiu Qu
143	Huai'an First People's Hospital	Eastern China	Jiangsu	Huai'an	Shuren Ma
144	Hunan Changsha County First People’s Hospital	Central China	Hunan	Changsha	Siding Wang
145	Li County Hospital of Traditional Chinese Medicine	Central China	Hunan	Changde	Songbai Li

146	The First Affiliated Hospital of Chongqing Medical University	Southwest China	Chongqing	Chongqing	Suxin Luo
147	Nanchong Central Hospital	Northwest China	Sichuan	Nanchong	Tao Liu
148	Ningjin People's Hospital	Eastern China	Shandong	Dezhou	Tao Zhang
149	Guang'an People's Hospital	Southwest China	Sichuan	Guang'an	Tian Tuo
150	Navy General Hospital	Northern China	Beijing	Beijing	Tianchang Li
151	Xiangya Hospital Central South University	Central China	Hunan	Changsha	Tianlun Yang
152	Gongyi people's hospital	Central China	Henan	Zhengzhou	Tianmin Du
153	Guangzhou Red Cross Hospital	Southern China	Guangdon g	Guangzhou	Tongguo Wu
154	Dongfeng Hospital	Northeast China	Jilin	Liaoyuan	Wei Liu
155	Zhejiang Provincial Hospital of TCM	Eastern China	Zhejiang	Hangzhou	Wei Mao
156	The First People's Hospital of Longquanyi District	Southwest China	Sichuan	Chengdu	Wei Tuo
157	The First Affiliated Hospital of Guangzhou Medical College	Southern China	Guangdon g	Guangzhou	Wei Wang
158	The Third Xiangya Hospital of Central South University	Central China	Hunan	Changsha	Weihong Jiang
159	The First Affiliated Hospital of Wenzhou Medical University	Eastern China	Zhejiang	Wenzhou	Weijian Huang
160	Affiliated Hospital of Qinghai University	Northwest China	Qinghai	Xining	Weijun Liu
161	Jianshui County People's Hospital	Southwest China	Yunnan	Honghe	Weiqing Fan
162	The Second Affiliated Hospital of Soochow University	Eastern China	Jiangsu	Suzhou	Weiting Xu
163	Teda International Cardiovascular Hospital	Northern China	Tianjin	Tianjin	Wenhua Lin
164	Wuhan Asia Heart Hospital	Central China	Hubei	Wuhan	Xi Su
165	Shanghai Jiading District Center Hospital	Eastern China	Shanghai	Shanghai	Xia Chen
166	Guangxi Hengxian County People's Hospital	Southern China	Guangxi	Nanning	Xianan Zhang

167	The Second Hospital of Hebei Medical University	Northern China	Hebei	Shijiazhuang	Xianghua Fu
168	The First Affiliated Hospital of Soochow University	Eastern China	Jiangsu	Suzhou	Xiangjun Yang
169	Changhai Hospital of Shanghai	Eastern China	Shanghai	Shanghai	Xianxian Zhao
170	Affiliated Hospital of Yan'an University	Northwest China	Shaanxi	Yan'an	Xiaochuan Ma
171	The First People's Hospital of Jining	Eastern China	Shandong	Jining	Xiaofei Sun
172	Longhui County People's Hospital	Central China	Hunan	Shaoyang	Xiaojun Wang
173	Tonglu First People's Hospital	Eastern China	Zhejiang	Hangzhou	Xiaolan Li
174	Xinmi people's hospital	Central China	Henan	Zhengzhou	Xiaolei Li
175	Zunhua People's Hospital	Northern China	Hebei	Tangshan	Xiaoli Yang
176	West China Hospital of Sichuan University	Northwest China	Sichuan	Chengdu	Xiaoping Chen
177	The Central Hospital of Taiyuan	Northern China	Shanxi	Taiyuan	Xiaoping Chen
178	Datong City Second People's Hospital	Northern China	Shanxi	Datong	Xiaoqin Zhang
179	The Second Affiliated Hospital to Nanchang University	Eastern China	Jiangxi	Nanchang	Xiaoshu Cheng
180	Yuzhong County People's Hospital	Northwest China	Gansu	Lanzhou	Xiaowei Peng
181	Qinyang People's Hospital	Central China	Henan	Jiaozuo	Xiaowen Ma
182	Hebei General Hospital	Northern China	Hebei	Shijiazhuang	Xiaoyong Qi
183	Yutian Hospital	Northern China	Hebei	Tangshan	Xiaoyun Feng
184	The Third Affiliated Hospital of Guangzhou Medical College	Southern China	Guangdong	Guangzhou	Ximing Chen
185	Chongqing Hechuan District People's Hospital	Southwest China	Chongqing	Chongqing	Xin Tang
186	The First Affiliated Hospital of Wannan Medical College	Eastern China	Anhui	Wuhu	Xingsheng Tang
187	Inner Mongolia People's Hospital	Northern China	Inner Mongolia	Hohhot	Xingsheng Zhao

188	Ledong Second People's Hospital	Southern China	Hainan	Ledong	Xiufeng Chen
189	Wuxi Xishan People's Hospital	Eastern China	Jiangsu	Wuxi	Xudong Li
190	Tangdu Hospital of The Fourth Military Medical University	Northwest China	Shaanxi	Xi'an	Xue Li
191	Shanghai East Hospital Affiliated to Tongji University	Eastern China	Shanghai	Shanghai	Xuebo Liu
192	Beijing Fangshan District First Hospital	Northern China	Beijing	Beijing	Xuemei Peng
193	The General Hospital of Shenyang Military Region	Northeast China	Liaoning	Shenyang	Yaling Han
194	Xiamen Cardiovascular Disease Hospital	Eastern China	Fujian	Xiamen	Yan Wang
195	Tieli People's Hospital	Northeast China	Heilongjiang	Yichun	Yanbo Niu
196	Dianjiang People's Hospital	Southwest China	Chongqing	Chongqing	Yang Yu
197	The First Hospital of Jilin University	Northeast China	Jilin	Changchun	Yang Zheng
198	The Second Affiliated Hospital of Qiqihar Medical Hospital	Northeast China	Heilongjiang	Qiqihar	Yanli Wang
199	General Hospital of Guangzhou Military Command	Southern China	Guangdong	Guangzhou	Yanlie Zheng
200	Fujian Provincial Hospital	Eastern China	Fujian	Fuzhou	Yansong Guo
201	The First Affiliated hospital of Dalian Medical University	Northeast China	Liaoning	Dalian	Yanzong Yang
202	The First People's Hospital of Changde	Central China	Hunan	Changde	Yi Huang
203	Tianjin Chest Hospital	Northern China	Tianjin	Tianjin	Yin Liu
204	Hunan Provincial People's Hospital	Central China	Hunan	Changsha	Ying Guo
205	Longmen People's Hospital	Southern China	Guangdong	Huizhou	Yingchao Luo
206	People's Hospital of Yuxi City	Southwest China	Yunnan	Yuxi	Yinglu Hao
207	The First Affiliated Hospital of China Medical University	Northeast China	Liaoning	Shenyang	Yingxian Sun
208	The People's Hospital of Guangxi Zhuang Autonomous Region	Southern China	Guangxi	Nanning	Yingzhong Lin

209	The First Teaching Hospital of Xinjiang Medical University	Northwest China	Xinjiang	Urumchi	Yitong Ma
210	Dazhou Central Hospital	Northwest China	Sichuan	Dazhou	Yong Guo
211	Mingguang People's Hospital	Eastern China	Anhui	Chuzhou	Yong Li
212	Baogang Hospital	Northern China	Inner Mongolia	Baotou	Yongdong Li
213	Jiangsu Binhai County People's Hospital	Eastern China	jiangsu	Yancheng	Yonglin Zhang
214	The Fourth Affiliated Hospital of China Medical University	Northeast China	Liaoning	Shenyang	Yuanzhe Jin
215	First Affiliated Hospital of Harbin Medical University.	Northeast China	Heilongjiang	Harbin	Yue Li
216	Sihui People's Hospital	Southern China	Guangdong	Zhaoqing	Yuehua Huang
217	Tianjin Medical University General Hospital	Northern China	Tianjin	Tianjin	Yuemin Sun
218	Qian'an People's Hospital	Northern China	Hebei	Tangshan	Yuheng Yang
219	Zhalantun People's Hospital	Northern China	Inner Mongolia	Hulunbeier	Yuhua Zhu
220	Longjiang First People's Hospital	Northeast China	Heilongjiang	Qiqihar	Yuhuan Shi
221	The Second Affiliated Hospital of Zhengzhou University	Central China	Henan	Zhengzhou	Yulan Zhao
222	Nanfang Hospital of Southern Medical University	Southern China	Guangdong	Guangzhou	Yuqing Hou
223	The First Affiliated Hospital to Nanchang University	Eastern China	Jiangxi	Nanchang	Zeqi Zheng
224	Cangzhou Central Hospital	Northern China	Hebei	Cangzhou	Zesheng Xu
225	The Central Hospital of Shaoyang	Central China	Hunan	Shaoyang	Zewei Ouyang
226	Yulong Hospital	Southwest China	Yunnan	Lijiang	Zeyuan He
227	Affilioted Hospital of North Sichuan Medical College	Northwest China	Sichuan	Nanchong	Zhan Lv
228	The People's Hospital of Liaoning Province	Northeast China	Liaoning	Shenyang	Zhanquan Li
229	The First Affiliated Hospital of Jiamusi University	Northeast China	Heilongjiang	Jiamusi	Zhaofa He

230	Tangshan Gongren Hospital	Northern China	Hebei	Tangshan	Zheng Ji
231	The First Affiliated Hospital of Lanzhou University	Northwest China	Gansu	Lanzhou	Zheng Zhang
232	The Third Hospital of Shijiazhuang	Northern China	Hebei	Shijiazhuan g	Zhenguo Ji
233	Huaibei Miners General Hospital	Eastern China	Anhui	Huaibei	Zhenqi Su
234	Wuxi People's Hospital	Eastern China	Jiangsu	Wuxi	Zhenyu Yang
235	Linyi People's Hospital	Eastern China	Shandong	Linyi	Zhihong Ou
236	Jiangsu Province Hospital	Eastern China	Jiangsu	Nanjing	Zhijian Yang
237	The Second Hospital of Shanxi Medical University	Northern China	Shanxi	Taiyuan	Zhiming Yang
238	The Affiliated Hospital of Xuzhou Medical College	Eastern China	Jiangsu	Xuzhou	Zhirong Wang
239	Southwest Hospital, Third Military Medical University	Southwest China	Chongqing	Chongqing	Zhiyuan Song
240	Zhijin People’s Hospital	Southwest China	Guizhou	Bijie	Zhongshan Wang
241	The First Affiliated Hospital of Xi’an Jiaotong University	Northwest China	Shaanxi	Xi'an	Zuyi Yuan

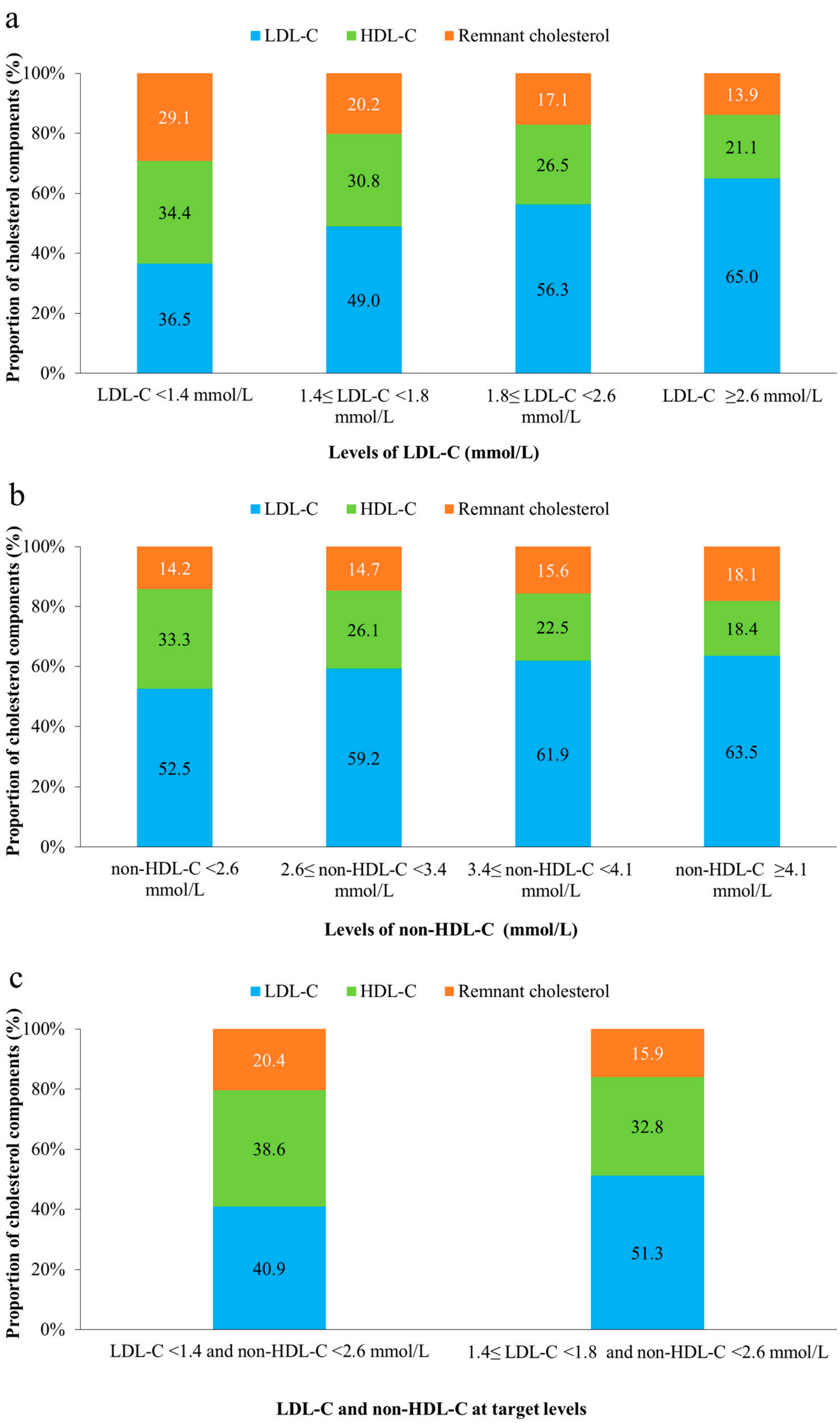

Supplementary Figure S1.(a) Proportion of different cholesterol components in total cholesterol among patients with different LDL-C levels; (b) Proportion of different cholesterol components in total cholesterol among patients with different non-HDL-C levels; (c) Proportion of different cholesterol components in total cholesterol among patients with LDL-C and non-HDL-C at target levels.
LDL-C, low-density lipoprotein cholesterol; non-HDL-C, non-high-density lipoprotein cholesterol.
